# Effective Coverage of Modern Contraceptive Use in Ethiopia: An Ecological Linking Analysis of Service Provision Assessment and National Health Equity Surveys

**DOI:** 10.3390/ijerph21121570

**Published:** 2024-11-26

**Authors:** Misrak Getnet, Samson Gebremedhin, Dessalegn Y. Melesse, Melinda K. Munos, Elizabeth A. Hazel, Yohannes D. Wado, Arega Zeru, Alemayehu Worku

**Affiliations:** 1 Health System and Reproductive Health Research Directorate, Ethiopian Public Health Institute, Addis Ababa 1242, Ethiopia; 2 School of Public Health, College of Health Sciences, Addis Ababa University, Addis Ababa 9086, Ethiopia; 3 Institute for Global Public Health, Department of Community Health Sciences, University of Manitoba, Winnipeg, MB R3E 0T6, Canada; 4 Johns Hopkins Bloomberg School of Public Health, Baltimore, MD 21205, USA; 5 African Population and Health Research Center, Nairobi P.O. Box 10787-00100, Kenya

**Keywords:** family planning, modern methods, readiness, effective coverage, quality, Ethiopia

## Abstract

The increase in contraceptive prevalence rate (crude coverage) in Ethiopia over the past two decades does not necessarily reflect service quality, and although the proportion of women with unmet needs has decreased, it remains unacceptably high. Hence, this study aimed to estimate the effective coverage (EC) of modern contraceptive methods in Ethiopia, considering the quality of care. We used nationally representative surveys, such as health facility surveys (Ethiopia Service Provision Assessment, 2021/22) and household surveys (National Health Equity Survey, 2022/2023). The descriptive analysis and ecological linking of the two surveys were used to assess the relationship between service quality and utilization among married/in union women in need of limiting or spacing children. In 2022, about 78% of health facilities in Ethiopia were ready to provide Family Planning (FP) services using modern contraceptive methods. Met FP need was 48%, with the quality of services assessed at 36%. After accounting for both service quality and readiness, Ethiopia’s effective coverage of family planning services using modern methods was estimated at 16%, with the highest coverage in the Sidama region (21%) and the lowest in the Somali region (2%). The EC of FP services in Ethiopia was low, largely attributed to the poor overall quality of the FP services provided. It is therefore important to ameliorate the quality of FP services in the country.

## 1. Introduction

Enhanced access to family planning services plays a crucial role in promoting reproductive health (RH), empowering individuals, and contributing to sustainable development [1]. Globally, the trend of FP services underscores both progress and persistent challenges. Although the use of modern contraception has increased in recent decades, significant geographic disparities in coverage, along with ongoing issues related to service quality, remain major challenges across various countries [2,3,4,5].

Over the past two decades, Ethiopia has experienced a significant increase in contraceptive prevalence, with coverage rising from 8.0% in 2000 to 45.9% in 2022 [6]. Several factors might have contributed to the increase in contraceptive prevalence in Ethiopia, including government initiatives promoting FP, improved access to contraception, and enhanced public awareness. Additionally, the expansion of health infrastructure and training for service providers have made modern contraceptives more accessible, especially in rural areas [7,8,9,10] supporting the broader goals of reducing unwanted pregnancies and enhancing public health. However, reports have indicated that the surge in modern contraceptive use did not align with a concurrent enhancement in the quality of FP counseling and other related services [3,7,8] perhaps contributing to the low effective coverage of FP highlighted in previous studies [3,11].

Household surveys are commonly used as the primary data source to assess health intervention coverage, primarily in developing countries, playing a key role in prioritizing, planning, and evaluating interventions at global, national, and sub-national levels. Nevertheless, these surveys and traditional coverage measures do not incorporate measures for the quality of the services provided [11]. To better understand the quality of services at health facilities from which individuals seek care and their impact on health outcomes, effective coverage cascades have been proposed. The study by Amouzou et al. defined the cascade groups as components of coverage organized in a stepwise pattern [12]. They also defined effective coverage as “the proportion of individuals experiencing health gains from a service among those who need the service” [12].

The main objective of this study was to estimate the EC of FP services using data from two nationally representative surveys, the Ethiopia Service Provision Assessment (ESPA)—a health facility survey that collects information on service availability, readiness of services, and quality of care measures within the health system—and the National Health Equity Survey (NHES)—a household survey designed to measure health equity in access to resources, service utilization, and health outcomes. Both were conducted between 2021 and 2023. The study is motivated by two factors; first, there is a notable scarcity of global and Ethiopia-specific research on the EC of FP services, with only one prior study conducted using data from the ESPA 2014 and Ethiopian Demographic and Health Survey (EDHS; 2016) [6,13] Since then, COVID-19 has impacted the health system, creating a significant gap in data from 2019 to 2024 and highlighting the need for updated research. Second, while the previous study focused only on quality-adjusted coverage, this study expands the methodology and included both service readiness- and quality-adjusted coverage. Furthermore, the Ethiopian government’s FP 2030 commitment aims to improve financing, ensure contraceptive security, enhance access for youth, and the quality of FP counseling, which was previously rated at just 30% [7].

## 2. Method

### 2.1. Study Setting

Ethiopia has eleven regions and two city administrations (Addis Ababa and Dire Dawa). The regions are Afar, Amhara, Benishangul-Gumuz, Central Ethiopia, Gambela, Harari, Oromia, Sidama, Somali, Southwest Ethiopia, and Tigray. This study focused on diverse populations in 10 regional states and 2 city administrations to assess the EC of FP services. The regions included in the study are Afar, Amhara, Oromia, Somali, Southern Nations Nationalities and People (SNNP), Gambela, Southwest Ethiopia, Harari, and Sidama, along with the Dire Dawa and Addis Ababa city administrations. The Tigray region was excluded from the analysis due to a lack of data caused by the ongoing conflict during the data collection period for both surveys. Although NHES later collected data from Tigray, we did not include it in this study because ESPA did not collect data in the region. Additionally, the Central Ethiopia region was not separated from SNNP during the data collection period. Our target population included women of reproductive age who are currently using or in need of FP services, as well as health facilities delivering these services. This approach allowed for a detailed analysis of both the readiness and quality of FP across the country.

### 2.2. Data Source

The two main surveys utilized in this study are ESPA 2022 and NHES 2022/2023. The ESPA data included information from health facilities offering FP services, health workers providing FP services, and clients attending health facilities for FP and leaving with a contraceptive method. A total of 1044 health facilities, comprising public and private hospitals combined (*n* = 350), health centers (*n* = 263), clinics (*n* = 185), and health posts (*n* = 246), were identified as providing FP services. Data from 2572 FP clients who were observed while receiving the service and had exit interviews before they left the facilities were used for the analysis.

The NHES is a nationally representative household survey conducted in 2022/2023 using a two-stage cluster sampling design. Women aged 15–49 in the sampled households were interviewed. Interviews included questions about the need for FP, the use of FP methods, and where the method was obtained. The survey sampled 8492 households and completed interviews successfully with 8429 households. More detailed information on the methodology and sample size of these surveys are available elsewhere [14].

### 2.3. Data Collection Method

For the facility assessment, data is sourced from the 2021–2022 Ethiopia SPA survey, which collected information on various aspects of care quality through multiple questionnaires. These included an inventory questionnaire assessing service availability and facility features; health worker interviews capturing data on duties, training, and demographics; and observation checklists documenting consultations, and exit interviews, gathering clients’ perceptions and demographic information. The survey observed up to 5 consultations per health worker and 15 per service, covering 1044 facilities offering family planning services. Additionally, household-based equity surveys conducted in 2022 included women aged 15–49, using multi-stage cluster sampling to provide nationally representative data. Detailed information on the data collection methods can be found in the referenced reports [14,15].

## 3. Measures

### 3.1. Target Population

The target population was women in need of FP services (women aged 15–49, who were either married or in union and expressed a desire to either space or limit childbearing and were not pregnant) or women with a met need (women aged 15–49, who were using a contraceptive method at the time of the survey).

### 3.2. Service Contact

The proportion of the target population who had contact with a family planning facility for any reason.

### 3.3. Met Need for FP Using a Modern Method

The proportion of the target population using a modern contraceptive method.

### 3.4. Readiness-Adjusted Coverage

The proportion of the target population currently using modern contraceptives obtained from the health facilities, scaled by the facility’s readiness to provide FP.

### 3.5. Quality-Adjusted Coverage

The proportion of the target population currently using modern contraceptives obtained from the health facilities, scaled by the quality of the facility’s provided FP.

### 3.6. Effective FP Coverage

We used quality-adjusted FP coverage as a proxy measure of the effective coverage, as recommended by the “Effective coverage Think Tank Group” [12].

### 3.7. Readiness of the Facilities

Four key domains listed by the World Health Organization (WHO) Service Availability and Readiness Assessment (SARA) guidelines to measure the structural/readiness of quality of the service were used [16], and these include the availability of modern contraceptives, qualified staff and guidelines, equipment, and stocked commodities. A total of 10 tracer items across these four domains were used to compute readiness scores. Table 1 provides further details.

### 3.8. Modern Contraceptives Methods

The modern contraceptive methods included in this study are pills, injectables, implants, male condoms, intrauterine contraceptive devices, and emergency contraceptive pills.

### 3.9. Process Quality

The process quality measure was defined as the percentage of the recommended clinical actions that were carried out during clinical visits. We identified 24 clinical actions based on the WHO FP handbook, the national guideline for FP, and previous studies [3]. These actions were uniformly weighted, and the quality of services was quantified by the percentage of these recommended actions completed during the observed visits. A further detailed list of these clinical actions is provided in Supplementary Materials (Supplementary Table S1).

### 3.10. Data Management and Analysis

We used NHES to identify the target population and to calculate service contact, met need for FP using a modern method, and facility-based met need at national and regional levels. Readiness scores were calculated from the SPA survey, measuring the structural quality variables. To assess the readiness structural quality domains listed in WHO’s SARA 2015 guideline, [17] was used. These include (1) service availability, (2) staff and guidelines, (3) equipment and supplies, and (4) contraceptive commodities and supplies. The tracer items for staff and guidelines are the national FP service guidelines or any other service guideline, and at least one staff member being trained in FP. The blood pressure apparatus is the only equipment to be used as the tracer item for the equipment and supplies domain. The contraceptive commodities are the combined estrogen–progesterone oral contraceptive pills (COC), progestin-only contraceptive pills (POP), injectable contraceptives, male condoms, intrauterine contraceptive devices (IUCD), and implants.

In ESPA, 2572 FP clients were observed receiving the service, marking the highest number compared to previous findings analyzed using observational data [3,4]. Therefore, we used process quality to measure the effective coverage of FP services.

The process quality measure for FP was defined as the percentage of recommended clinical actions that were carried out during clinical visits. We identified 24 clinical actions based on the WHO FP handbook, the national guideline for FP, and previous studies [3]. These actions were uniformly weighted, and the quality of service was quantified by the percentage of these recommended actions completed during observed visits. A further detailed list of these clinical actions is provided in Supplementary Materials (Supplementary Table S1).

The quality of service is the percentage of recommended clinical actions performed during observation visits. We measured the quality of care using data from the SPA2021–22 to evaluate the quality of FP services based on the WHO handbook, the Ethiopian National FP Guidelines, and earlier research. A total of 24 clinical actions were chosen from the SPA dataset. Therefore, the process component had 24 binary factors that assessed the healthcare providers’ compliance with FP service standards. The overall quality index of FP services was computed using technical quality, calculated as a percentage of all recommended actions completed, with all actions given equal weight.

The quality of care for FP services using the process of FP services was calculated using the following formula:

(1) $$Process\;quality\;index=\frac{Total\;number\;of\;‘yes’\;responses\;to\;process\;component\;parameters}{Total\;number\;of\;parameters\;in\;the\;process\;component}\times 100$$

Finally, we calculated the quality-adjusted level for FP services in Ethiopia by multiplying FP met need by a quality score calculated from the content of care provided.

The following formula was used to calculate the effective coverage of FP services [18]:

(2) EC=(CC×Q)

where ECij is EC for individual regions (i) and intervention j, Q is the observed quality, and CC is the crude coverage of FP met need.

To estimate the readiness- and quality-adjusted coverage, we used an ecological linking approach, where stratum average readiness and quality scores were multiplied by the stratum coverage (met need using modern contraceptives sourced from a health facility). Strata were defined by administrative area (region) and facility type. This yielded readiness- and quality-adjusted coverage estimates ranging from 0 (no coverage or quality) to 100% (perfect coverage and quality).

For the subnational analysis by geography, due to differences in the SPA and NHES datasets, SPA data from the Sidama and Southwest Ethiopia regions were merged and presented as Sidama, and Tigray was excluded from the analysis, as no data were collected due to the conflict during the data collection period. Overall, we followed specific steps to analyze this study. First, we developed a provider crosswalk, followed by defining the clinical actions and readiness domains. Next, we generated the quality of care metrics and the readiness of the facilities, and we linked the health facility survey with the population survey using an ecological analysis. Finally, we calculated the quality-adjusted coverage. We used STATA version 18 software to analyze the data.

### 3.11. Ethical Considerations

This study used secondary data from the ESPA and NHES surveys, for which consent was obtained from all participants. The datasets were requested online, and permission to use the data was granted by the Demographic Health Survey (DHS) program and Ethiopian Public Health Institute (EPHI). This research received ethical approval from the Addis Ababa University College of Health Sciences Institutional Review Board (IRB), with reference number 053/24/SPH.

## 4. Results

### 4.1. Sociodemographic Characteristics of Reproductive Age Women (NHES 2022/2023)

The sociodemographic characteristics of women interviewed in the 2022/2023 equity survey are described in Table 2. The mean age of the respondents was 29. The age group of 25–34 accounted for 30.6% of this population, with large representation in the 20–24 (17.9%) and 35–39 (16.8%) categories. There was a wide range of educational attainment; the largest group was uneducated (47.0%), followed by those with only primary education (34%). The majority of women (95.1%) were married or in union. Religion was diverse; the two most common faiths are Orthodox (46.8%) followed by Muslim (32.6%). A small minority of people (18.33%) were employed, while unemployment is substantial.

### 4.2. Demand Satisfied, Readiness and Readiness-Adjusted Coverage of FP Services in Ethiopia

Table 3 presents the demand satisfied, readiness, and readiness-adjusted coverage of FP services in Ethiopia. There were significant disparities in the demand satisfied for FP using modern contraceptive methods among the regions, with Sidama and Benishangul Gumuz having relatively high coverage rates of 65% and 59%, respectively, while regions like Somali and Afar have much lower coverage rates, 5% and 11%, respectively (Table 3). The national average stands at 48%.

The overall readiness of the facilities to provide FP services was 78%, with the facilities in Harari being 90% ready to provide the service compared to SNNP, where only 69% of the facilities were ready to provide the service.

Overall, the readiness-adjusted effective coverage of FP services in Ethiopia was 36.1%, with 95% confidence intervals (CIs) between 35.2% and 37.0% (Table 4). There was regional and locational variation in the readiness-adjusted coverage of FP services, with Sidama having the highest coverage (50%), followed by Amhara (44%). Additionally, facilities located in urban areas had a higher readiness-adjusted coverage compared to those in rural areas.

### 4.3. Recommended FP Care Clinical Actions

Out of the 24 recommended clinical actions, the data shows that 55% of the women observed received between 7 and 12 clinical actions (Table 4). However, a sizable portion of women (30%) received 0–6 clinical actions. Interestingly, the most commonly observed FP clinical actions were recording information on the client card (85.7%) and offering counseling on various FP method-related difficulties (83.6%) (Supplementary Figure S1). On the other hand, asking about STI symptoms (3.9%), evaluating the partner/relationship status (3.9%), and providing advice on the use of condoms for STI prevention (1.1%) were the least observed clinical actions. Overall, FP users receive 36% of the clinical actions that are advised.

### 4.4. Quality of Care in FP Services by Facility Type

**Figure 1:**
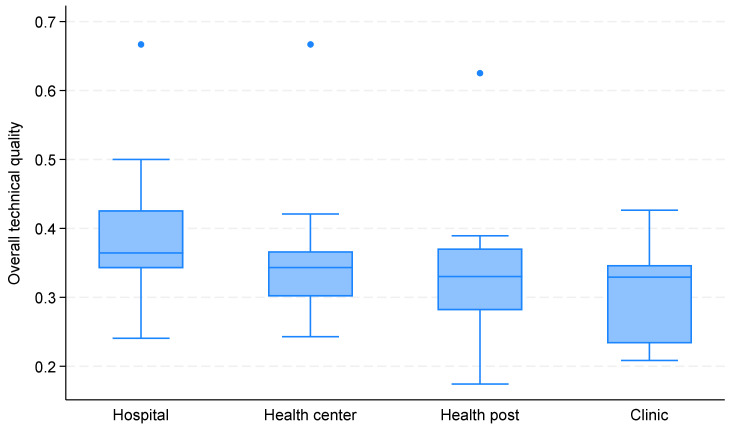
Quality of care in FP services by facility type.

Figure 1 shows the value of quality of care across the different health facility types. The overall quality of care was 33.8% (IQR: 30.1–37.1%). Hospitals had a higher quality of care compared to other institutions; their median score is 37.5%, IQR: 34.2–42.6%, and their upper range goes up to 84%. The quality of care in health centers follows, with a median of 33.3%, IQR: 30.1–36.7%. The quality of care in clinics and health posts was somewhat lower.

### 4.5. Quality-Adjusted Coverage of FP Services

Table 5 shows the summary of FP services offered in various Ethiopian regions. The data indicated that although most regions have a considerable met need for FP services (national average: 48.1%), the process quality-adjusted coverage is substantially lower, averaging only 15.5%. This shows a significant gap of 33 percent points between the demand satisfied for FP using a modern method and the process quality-adjusted coverage. Regions such as Sidama had a higher process quality-adjusted coverage (21%) compared to Afar and Somalia (4% and 2%, respectively), indicating a significant gap in the process quality-adjusted coverage between regions.

### 4.6. Effective Coverage of FP Services in Ethiopia

**Figure 2:**
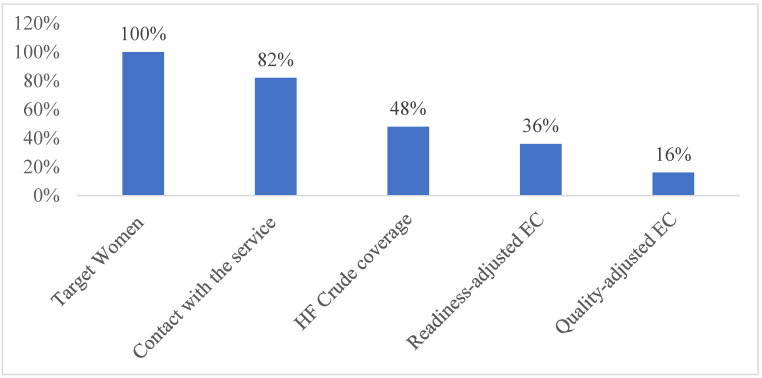
Cascade of effective coverage of FP services in Ethiopia.

Figure 2 illustrates the effective coverage cascade for FP services in Ethiopia. After adjusting for facility readiness and the process quality of the service, the readiness-adjusted FP coverage and the quality-adjusted FP coverage in Ethiopia were low, at 36.1% (95% CI: 35.2, 37.0) and 15.5% (95% CI: 15.1, 15.9), respectively.

### 4.7. Effective Coverage of FP Services in Ethiopia by Region and Location of the Facilities

**Figure 3:**
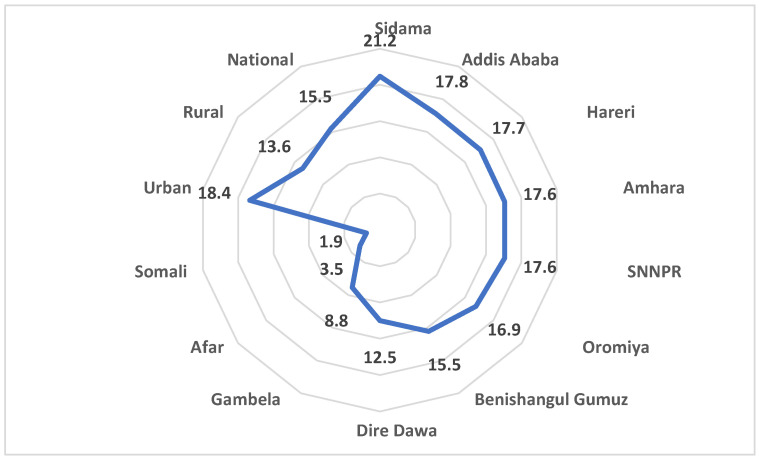
Effective coverage of FP services in Ethiopia by region and location of the facilities.

Figure 3 shows effective coverage of family planning services in Ethiopia. Overall Effective coverage of FP services in the country was 16%. There was a slight regional variation, with Sidama (21%) having the highest coverage, while the Somali and Afar regions had the lowest, with 2% and 4%, respectively. Effective coverage was also higher in the facilities located in urban areas compared to rural areas.

## 5. Discussion

The high coverage of essential health services does not always result in an impact on health outcomes. There should be an overall increase in the readiness of services and the quality of care [19,20]. We found that despite efforts to improve the quality of FP services in the country, there is still a significant gap in the overall coverage and quality of FP health services.

This study revealed that the process quality-adjusted coverage of FP services in Ethiopia was very low (16%), and it was lower than that estimated by a previous study [3]. This suggests that despite efforts to improve the quality of FP services in the country, there is still a significant gap between the demand satisfied for FP and the process quality-adjusted coverage of FP health services. The possible reasons for the difference from the previous study conducted on the effective coverage of FP services in Ethiopia are the following: The previous study used EDHS to measure met FP needs, while this study used a national health equity survey. In addition, the health system has been affected by the COVID-19 pandemic. The previous study was conducted before the pandemic. During the pandemic, there was also a chronic shortage of FP commodities brought on by a variety of factors, such as a lack of funding, a halt to donor support, delayed procurement, and issues with the management of the supply chain throughout the health system [21].

We found a large gap between average facility readiness to provide FP services (78.0%) and the average process quality of FP services (33.8%). The finding of this study is also low compared to the effective coverage of modern FP services in other African countries. For instance, the effective coverage of FP services in Kenya was 51% in 2014 [22]. It is also low compared to a previous study conducted in Ethiopia, where the effective coverage of FP services and the crude coverage of met FP needs was 21.7% and 60.6%, respectively [3].

The low effective coverage and poor quality of care can negatively affect FP program outcomes, despite the high facility readiness. This shows that the idea of improving service availability alone is not sufficient without ensuring service quality [22].

Looking at effective coverage by region, we found that the Sidama region had higher effective coverage of FP services, with several other regions close behind. However, a few regions including Afar, Somali, and Gambela had much lower levels of quality-adjusted coverage. These findings indicate that some regions are better at meeting the standards and providing a fuller range of services, but all regions have room for improvement. Improving comprehensive FP service delivery in Ethiopia requires addressing regional differences in effective coverage.

According to the national average, less than half of Ethiopian women’s FP needs are currently met. These differences highlight the importance of implementing targeted interventions to increase access to FP services for disadvantaged areas. A significant proportion of women received less than half of the recommended clinical actions. This suggests possible shortcomings in the quality of FP treatment, which could affect the effectiveness of programs and women’s overall health. These gaps could be partially filled by implementing quality-of-care improvement strategies, such as training health providers, to ensure they provide the necessary quality of care to clients.

The strength of this study is that we used the most recent ESPA data by incorporating the national health equity survey dataset to obtain population-level data. In addition, the conclusions are based on clinical care observation, which is more comprehensive than written data and is not subject to recall bias. Furthermore, the number of observed women during clinical care is the highest compared to previous studies. Hence, it is crucial to ascertain the effective coverage of FP services in the country. Moreover, this study utilized both structural and process quality measures to assess the effective coverage of modern FP services. However, the study did not include the two components of the effective coverage cascade (user adherence-adjusted and outcome-adjusted coverage) in estimating effective coverage. In addition, we were unable to use the standard definition of demand satisfied, because the NHES did not collect data to identify women who were infecund. This may have resulted in slightly lower estimates of the demand satisfied and effective coverage relative to other studies. However, our estimates were still quite low, so we do not think this had a large impact on our results. The NHES also collected data from the oldest women, which may not be representative of younger women and girls.

## 6. Conclusions

Monitoring facility assessments and household surveys provides critical information for FP programs. Overall, the effective coverage of FP services in Ethiopia was low, largely attributed to the lower readiness of health facilities and the poor quality of FP services provided. The possible health benefits of using these services are reduced by the quality. It is therefore important to improve both the readiness and quality of FP services in the country. In addition, addressing urban–rural disparities is also crucial in alleviating problems related to the quality of care and resource allocation in the two areas.

## Figures and Tables

**Table 1:** Tracer items used to measure service readiness in the SPA 2021/22 [17].

Domain	Item	Variable Name
Availability	Modern contraceptive mix	Facility provides/refers to all modern methods: pills, injectables, implants, condoms, IUCD, and ECPs
Staff and guidelines	Guidelines on FP	National FP service guidelines or any other service guidelines on FP available and observed
	FP checklists or job aid	
	Trained staff on FP	Any provider of FP services in this facility trained in FP at any time during the past 24 months
Equipment	BP cuff	BP cuff observed/functioning anywhere in facility
Commodities stocks	COC	At least 1 valid dose observed available on the day of assessment
	Progestin OC	
	Injectable contraceptives	
	Male condoms	
	Implant	
	IUCD	

Note: FP = family planning, BP = blood pressure, COC = combined estrogen-progesterone oral contraceptive pills, IUCD = intrauterine contraceptive devices, OC = oral contraceptive pills, ECPs = emergency contraceptive pills.

**Table 2:** Sociodemographic characteristics of reproductive age women, N = 6625 (NHES 2022/2023).

Stratifies	Group	Unweighted N (%)	Weighted N (%)
Age	15–19	163 (2.5)	402.8 (2.58)
	20–24	1227 (18.5)	2800.9 (17.91)
	25–29	2109 (31.8)	4913.7 (31.41)
	30–34	1508 (22.8)	3411.2 (21.81)
	35–39	1071 (16.2)	2636.5 (16.85)
	40–44	368 (5.6)	1027.5 (6.57)
	45–49	179 (2.7)	450.1 (2.88)
Educational status	No education	2804 (42.3)	7383.3 (47.2)
	Primary education	2257 (34.1)	5375.5 (34.4)
	Secondary education	897 (13.5)	1825.4 (11.7)
	More than secondary	667 (10.1)	1058.7 (6.8)
Marital status	In a union	14 (0.2)	63.7 (0.4)
	Married	6611 (99.8)	15,579.0 (99.6)
Religion	Orthodox	2427 (36.6)	7580.6 (48.5)
	Protestant	1874 (28.3)	2893.5 (18.5)
	Muslim	2279 (34.4)	5095.1 (32.6)
	Catholic	41 (0.6)	64.7 (0.4)
	Others	4 (0.1)	8.9 (0.1)
Region	Sidama	1239 (18.7)	1235.5 (7.9)
	Oromiya	804 (12.1)	6330.4 (40.5)
	Amhara	801 (12.1)	4480.7 (28.6)
	SNNPR	688 (10.4)	1983.5 (12.7)
	Addis Ababa	656 (9.9)	819.9 (5.2)
	Benishangul Gumuz	499 (7.5)	119.8 (0.8)
	Afar	476 (7.2)	134.7 (0.9)
	Gambela	452 (6.8)	60.3 (0.4)
	Dire Dawa	421 (6.4)	59.0 (0.4)
	Hareri	310 (4.7)	28.8 (0.2)
	Somali	279 (4.2)	390.3 (2.5)
Wealth Index	Poorest	1378 (20.8)	4437.5 (28.4)
	Poorer	1344 (20.3)	3886.7 (24.9)
	Middle	1276 (19.3)	3003.7 (19.2)
	Richer	1261 (19.0)	2283.12 (14.6)
	Richest	1366 (20.6)	2031.9 (13.0)
Total		6625 (100)	15,643 (100)

**Table 3:** Demand satisfied, and readiness and readiness-adjusted coverage of FP in Ethiopia, ESPA and NHES.

Region	Demand Satisfied for FP		Readiness Scores (% and 95% CI)		Readiness-Adjusted Coverage (%)	
	%	95% CI	%	95% CI	%	95% CI
Afar	11.3	8.8, 14.5	0.74	72.0, 76.1	8.0	5.9, 10.1
Amhara	58.4	0.55, 62.0	0.77	76.1, 78.6	44.2	41.5, 46.9
Oromiya	50.6	47.1, 54.0	0.75	74.6, 76.3	37.2	34.6, 39.8
Somali	4.7	2.7, 7.9	0.83	80.2, 86.3	3.9	1.8, 5.9
B-Gumuz	59.3	54.9, 63.5	0.80	78.0, 81.1	45.6	42.1, 49.1
SNNP	50.3	46.6, 54.0	0.69	67.7, 70.5	34.0	31.4, 36.7
Gambela	33.0	28.8, 37.4	0.80	77.6, 82.1	25.2	21.7, 28.6
Harari	48.1	42.5, 53.6	0.90	89.7, 91.1	38.0	33.3, 42.8
Addis Ababa	52.0	48.1, 55.7	0.87	85.5, 87.5	38.7	35.6, 41.8
Dire Dawa	38.5	33.9, 43.2	0.87	85.3, 88.1	30.2	26.4, 34.2
Sidama	64.8	62.1, 67.4	0.77	76.3, 77.7	3.9	1.8, 5.9
Location						
Urban	54.0	52.1, 55.9	0.83	82.6, 83.6	42.0	40.4, 43.5
Rural	44.2	42.6, 45.7	074	73.8, 75.0	32.2	30.9, 33.3
National	48.1	46.9, 49.3	0.78	77.6, 78.6	36.1	35.2, 37.0

**Table 4:** Number of recommended FP care clinical actions received by women, SPA 2021–22, Ethiopia.

Intervention	Number of Recommended Clinical Actions Received by Women					
FP (score out of 24)	Score (out of 24)	0–6	7–12	13–18	19–24	Total
	Number of women	764	1426	375	7	2572
	Percent of women received clinical action	29.7	55.4	14.9	0.3	100

**Table 5:** The summary of FP services offered in various Ethiopian regions.

Region	Demand Satisfied for FP Using a Modern Method (%)	Quality-Adjusted Coverage (%)	Absolute Gap Between Demand Satisfied and Quality-Adjusted Coverage (Percentage Points)
Afar	11.3	3.5	7.8
Amhara	58.4	17.6	40.8
Oromiya	50.6	17.0	33.6
Somali	4.7	2.0	2.7
Benishangul Gumuz	59.3	15.5	43.8
SNNP	50.3	17.7	32.6
Gambela	33.0	8.8	24.2
Harari	48.1	17.7	30.4
Addis Ababa	52.0	17.8	34.2
Dire Dawa	38.5	12.5	26.0
Sidama	64.8	21.2	43.6
National	48.1	15.5	32.6

## Data Availability

The ESPA data were obtained from the ICF International DHS Program (https://dhsprogram.com/data/new-user-registration.cfm) (accessed on 4 September 2023) and are accessible online. However, the NHES data supporting the findings are available upon request from EPHI.

## Supplementary Materials

The following supporting information can be downloaded at: https://www.mdpi.com/article/10.3390/ijerph21121570/s1, Figure S1: FP care quality in Ethiopia, SPA 2021–2022; Table S1: List of clinical actions.
