# Supplementary material for: Effective Coverage of Modern Contraceptive Use in Ethiopia: An Ecological Linking Analysis of Service Provision Assessment and National Health Equity Surveys
Source: Int J Environ Res Public Health. 2024 Nov 26;21(12):1570. doi: 10.3390/ijerph21121570 (PMC11675477; doi:10.3390/ijerph21121570)
Supplement: Supplementary file 1 [file ijerph-21-01570-s001.zip › ijerph-3276667 supplementary.pdf]

Supplementary Materials

Table S1: List of clinical actions

Item	N (%)
1. Asked: client age	795 (30.9)
2. Asked: number of living children	898 (34.9)
3. Asked: last delivery date	787 (30.6)
4. Asked: reproductive intentions	562 (21.9)
5. Asked: desired timing for birth of next child	594 (23.1)
6. Checked: current breastfeeding	396 (15.4)
7. Asked: menstrual regularity	746 (29.0)
8. Measured: client blood pressure	848 (33.0)
9. Measured: client weight	787 (30.6)
10. Asked: last menstrual period	1,069 (41.6)
11. Asked: STI symptoms	102 (4.0)
12. Asked: chronic illnesses	207 (8.1)
13. Checked: client's health card	1,898 (73.8)
14. Assured: confidentiality	688 (26.8)
15. Discussed: partner/ relation status	100 (3.9)
16. Asked: risk of STIs	112 (4.4)
17. Discussed: STIs prevention with condoms	28 (1.1)
18. Asked: if client has questions/concerns on FP	1,146 (44.6)
19. Wrote note: on client card	2,202 (85.6)
20. Used: visual aids	409 (15.9)
21. Discussed: follow up visit	1,826 (71.0)
22. Ensure: visual privacy	2,105 (81.8)
23. Ensure: auditory privacy	1,897 (73.8)
24. Counselled: at least on 1 issue on FP	2,149 (83.6)
**Over all technical quality**	**33.8**

Recommended FP care clinical actions

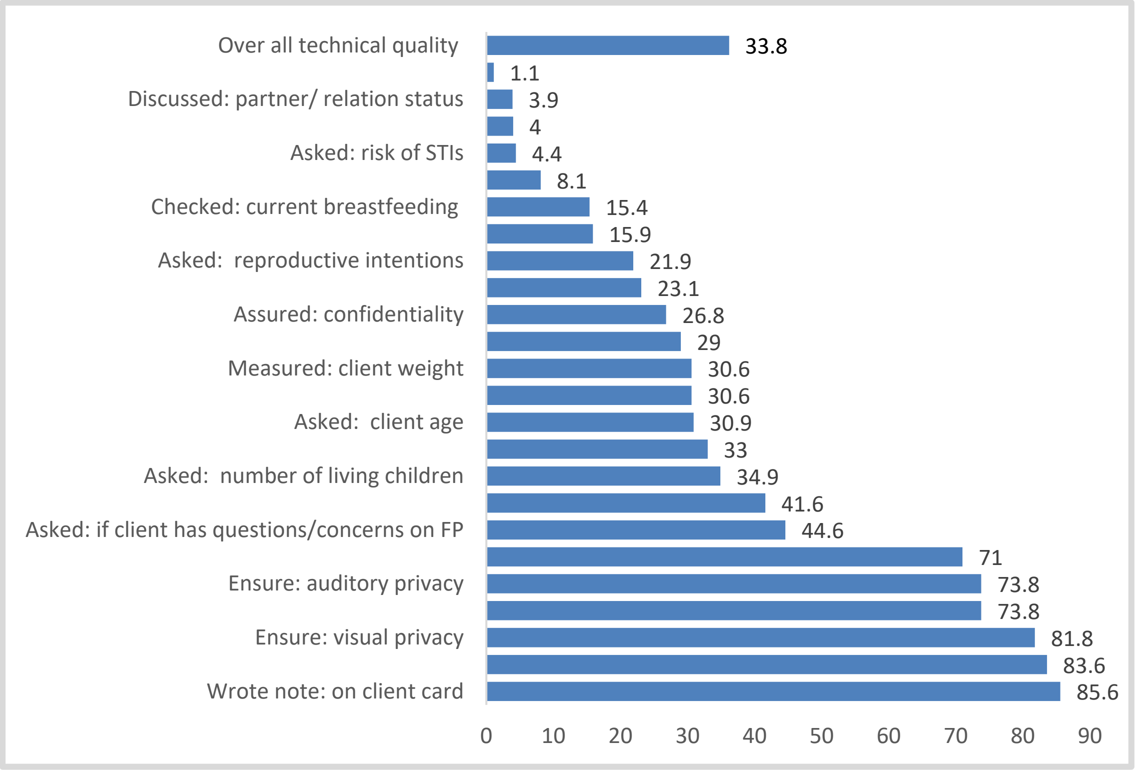

Figure S1: FP care quality in Ethiopia, SPA 2021-2022.
